# The Role of Serotonin in Singultus: A Review

**DOI:** 10.3389/fnins.2020.00629

**Published:** 2020-07-16

**Authors:** Georg A. Petroianu, Dietrich E. Lorke

**Affiliations:** ^1^College of Medicine and Health Sciences, Khalifa University, Abu Dhabi, United Arab Emirates; ^2^Herbert Wertheim College of Medicine, Florida International University, Miami, FL, United States

**Keywords:** singultus, hiccup, vagal maneuver, serotonin, aripiprazole, buspirone

## Abstract

The use of dopamine receptor blockers for chronic singultus treatment is based—at least partially—on circular thinking: chlorpromazine is FDA-approved for hiccups, chlorpromazine is a neuroleptic, neuroleptics are dopamine receptor blockers, and therefore hiccup is due to dopaminergic dysfunction. Chlorpromazine interacts with high affinity with a multitude of receptors and ion channels. This promiscuity is the basis for many of the therapeutic effects and adverse drug reactions of this drug. While an involvement of dopamine is certain, it is by no means clear that dopaminergic dysfunction is the hallmark of singultus. The common denominator of most remedies for transient hiccup is their ability to activate the vagus nerve. Both afferent and efferent vagal activity and the central integration of the Xth cranial nerve function are modulated, inter alia, via serotonergic mechanisms; beneficial (therapeutic) effects for hiccup are to be expected from serotonin (5-HT) receptor subtype ligands that enhance vagal activity. Taken together, it appears that the ability to increase vagus output is mainly associated with 5-HT_1A_, 5-HT_3_, and 5-HT_7_ agonists and with 5-HT_2C_ antagonists. The plausibility of the serotonergic singultus hypothesis is examined against available pharmacokinetic, pharmacodynamic, and clinical data for a number of drugs.

## Introduction

Hiccup (Latin, singultus) is generated by an involuntary contraction of the diaphragm followed by closure of the glottis. The inspired air meeting a closed glottis causes the typical hiccup sound. Hiccupping of extended duration can be incapacitating (Petroianu, 2019).

Most classifications use arbitrary time limits to categorize the phenomenon. Brief episodes of hiccupping are physiologic. The point of transition to a pathologic form is not well defined. The longer the duration of the hiccupping, the less amenable it will be to interventions. An episode lasting longer than a week is considered chronic while resistance to sequential therapy using three different drugs warrants the use of the label obstinate (Petroianu, 2019).

Hiccup is not a disease but a symptom. The situation most commonly encountered is that of hiccup of idiopathic origin. In this context, “idiopathic” describes one’s inability to demonstrate, rather than the absence of, an organic origin.

Probably only a few drugs in the **Physician’s Desk Reference** have not been tried in the therapy of singultus, and anyone who looks hard enough at the literature will be able to find anecdotal support for the use of almost any drug. In contrast, only a few drug categories (benzodiazepines, barbiturates, alcohol, and steroids) are well-established hiccup inducers (Petroianu, 2019).

Prevalence of chronic obstinate singultus was estimated in Germany in the 1990s at 1:10^3^–1:10^5^, with an overwhelming elderly male preponderance (Petroianu and Brunnengraber, 1992).

### Hiccups and Dopamine

The introduction of chlorpromazine into clinical practice in the early 1950s had a major impact on psychiatry. The drug revolutionized the discipline and established the field of psycho-pharmacology (Laborit et al., 1952; Ban, 2007). The success of chlorpromazine as an antipsychotic (neuroleptic) combined with the fact that it was far superior to the (very) few other central nervous system (CNS) drugs available at the time (morphine, hyoscine, and quinidine) led to its use for a multitude of conditions (Ey and Faure, 1956).

One of the conditions chlorpromazine was tested for was chronic (obstinate) hiccup, and positive case results were reported by various groups (Moyer et al., 1954; Stewart and Redeker, 1954; Davignon et al., 1955; Friedgood and Ripstein, 1955; Garipuy and Raymond, 1956; Guiang and Leones-Guiang, 1957).

The manufacturer Smith Kline and French advertised “*another dramatic use of Thorazine: to stop intractable hiccups (often after the first dose) in 56 out of 62 patients in seven different studies,”* and the United States Food and Drug Administration (FDA) approved chlorpromazine for the treatment of hiccups (Thorazine advertisements, 1954, 1955).

Chlorpromazine established itself as a successful hiccup treatment, even after attributing some of the reported success rate to a difficult-to-quantify placebo effect (Friedman, 1996).

While chlorpromazine efficacy for chronic hiccup treatment is generally accepted, the mechanism of action is unclear and not necessarily identical with the antipsychotic mechanism of action. Chlorpromazine has a rich pharmacology with at least if not greater affinity for a range of other targets.

This promiscuity is the basis for many of the therapeutic effects and adverse drug reactions (ADRs) of this drug. The antipsychotic usefulness of the drug is related to its ability to block dopamine and serotonin (5-HT) receptors. Among the more relevant ADRs to be named are orthostatic hypotension (α-adrenergic blockade), dry mouth, urinary retention, and other signs and symptoms of parasympathetic inhibition (muscarinic cholinergic blockade), Parkinson’s-like symptoms, decrease in libido and increase in plasma prolactin levels (dopaminergic blockade), sedation and weight gain (histaminergic blockade), weight gain, and anhedonia (5-HT_2C_ serotonergic blockade), and QT prolongation (inhibition of the *human ether-a-go-go-related gene* = hERG potassium channel). While the affinity of chlorpromazine for hERG channels is low (high K_i_), the ability to block other sites and induce ADRs is comparable or even higher than its affinity for the sites associated with the *antipsychotic* response.

The assumption that dopamine receptor blockers must be the pillar for treatment of hiccups is—at least partially—based on circular thinking: chlorpromazine is FDA-approved for hiccups, chlorpromazine is a typical neuroleptic, neuroleptics are dopamine receptor blockers, and therefore hiccup is a manifestation of dopaminergic dysfunction.

### Evidence for Dopaminergic Involvement

• Reports of dopaminergic agents (*amantadine, levodopa, pergolide, piribedil*, and *pramipexole*) inducing hiccups (Launois et al., 1993; Bagheri et al., 1999; Sharma et al., 2006).
• Reports of selective anti-dopaminergic agents (*haloperidol*) being able to control hiccups (Korczyn, 1971; Scarnati, 1979; Ives et al., 1985).

### Evidence Against Dopaminergic Involvement

• Reports of failure of anti-dopaminergic agents to control hiccups (Schuchmann and Browne, 2007).
• Reports of anti-dopaminergic agents (*perphenazine*) inducing hiccups (Miyaoka and Kamijima, 1999; Cheng et al., 2011).
• Reports of dopaminergic agonists (*amantadine, apomorphine, pergolide, pramipexole, piribedil, levodopa, ropinrole*) used to treat hiccup (Welsh, 1904; Garrick, 1917; Askenasy et al., 1988; Martinez-Ruiz et al., 2004; Sharma et al., 2006; Lester et al., 2007; Gerschlager and Bloem, 2009; Coletti Moja, 2010).
• Reports of failure of selective anti-dopaminergic agents (*haloperidol*) to control hiccups (Nishikawa et al., 2015).
• Discrepancies between the incidence of use of dopaminergic agonists and the incidence of hiccup, although these might be due to underreporting (Stegmeier-Petroianu and Petroianu, 2008; Miwa and Kondo, 2010).

Taken together, the evidence indicates that while an involvement of dopamine as a neurotransmitter in the hiccup reflex circuitry is certain, it is by no means clear that dopaminergic overactivity is the common denominator of hiccups, and therefore dopaminergic blockade must not necessarily be the main thrust of therapeutic attempts.

### Non-dopaminergic Therapies of Hiccup

Hiccupping is a physiologic occurrence during intrauterine life (Miller and Petroianu, 2016). It has been proposed that hiccup is an essential and universal primitive reflex that may recur, like other primitive reflexes, in adult life (Ingiulla, 1962; Dunn, 1977; Fuller, 1990; Steger et al., 2015). The suggested hiccup reflex arc consists of vagal, phrenic, and sympathetic afferents, a hiccup center in the upper spinal cord/brainstem region, and efferents that elicit a contraction of the diaphragm and the external intercostal muscles along the phrenic and intercostal nerves, as well as, immediately thereafter, a closure of the glottis via the vagus nerve, whose motor fibers travel with the recurrent laryngeal nerve to the larynx (Askenasy, 1992; Friedman, 1996; Steger et al., 2015).

In the adult, this primitive reflex is suppressed (Oshima et al., 1998; Straus et al., 2003). Reappearance is explained either by the loss of inhibition from hierarchically higher structures or by a surge in input from the periphery.

Many therapeutic strategies, with the GABA_*B*_ receptor agonist baclofen and the α_*2*_-δ ligands (gabapentin, pregabalin) being the most successful ones (Burke et al., 1988; Lance and Bassil, 1989; Ramirez and Graham, 1992; Guelaud et al., 1995; Petroianu et al., 1997, 2000; Petroianu, 1998; Jatzko et al., 2007), non-specifically reduce neurotransmitter release; their unquestionable success in chronic hiccup treatment does not, however, allow any inference as to the specific neurotransmitters and their receptors involved in the assumed hiccup reflex circuitry (black-box approach).

More contributory to the understanding of the pathophysiology is a look at therapies empirically established for the suppression of the so-called occasional or transient hiccup. These have recently been reviewed, and the author concludes that the common denominator of most, if not all of these homegrown remedies is their ability to activate the vagus nerve, as evidenced by their additional ability to terminate paroxysmal supraventricular tachycardias (Petroianu, 2013, 2015, 2020). Among the best-known “vagal maneuvers” are the oculo-cardiac reflex (Dagnini-Aschner), carotid sinus massage, the Valsalva maneuver, and ice ingestion. While usually effective in terminating bouts of acute hiccup, they are mostly ineffective in cases of hiccupping that have been present for an extended period, probably due to insufficiently sustained vagus nerve activation (Petroianu, 2015). The successful use of vagus nerve electrical stimulation for chronic intractable hiccups has been reported (Payne et al., 2005; Longatti et al., 2010) as have been failures of this approach (Grewal et al., 2018). The rationale for stimulating the left vagus nerve is that it innervates the AV node of the heart so as to have less of an effect on heart rate than the right vagus, which innervates the SA node (Carreno and Frazer, 2017).

### Nuclei of the Vagus Nerve

**Nucleus tractus solitarius** (NTS) receives general visceral afferent information. According to Jordan “*the NTS can be considered the brainstem equivalent of the dorsal horn*” (Jordan, 2005). The NTS is involved in a number of reflex mechanisms (gag, carotid sinus, cough, and vomiting reflex).

**Nucleus ambiguus** (NA) is in the medullary reticular formation. The nerve fibers originating from the NA are efferent visceral motor fibers that provide motor innervation for swallowing and phonation. This vagal nucleus innervates most striated muscles of the pharynx and larynx. The NA also contains the majority (≈90%) of preganglionic cholinergic parasympathetic neurons that innervate postganglionic parasympathetic neurons in the heart.

**Dorsal vagal nucleus** (DVN) sends parasympathetic visceral efferent fibers to thoracic and abdominal viscera.

The activation of cardiac vagal outflow by afferents involves a multi-synaptic pathway within the brainstem. Cardiorespiratory afferents terminate within the NTS. Neurotransmitters used by vagal afferents include peptides such as substance P and calcitonin gene-related peptide and the excitatory amino acid transmitter glutamate (Carreno and Frazer, 2017). Within this nucleus, the information is processed and integrated before passing to the output neurons located within the DVN and NA (Skinner et al., 2002; Jordan, 2005).

### Influence of Serotonin (5-HT) Upon Autonomic Nervous System Activity

5-HT interacts with the autonomic nervous system, in particular its parasympathetic component (vagus nerve) at several levels.

**FIGURE 1:**
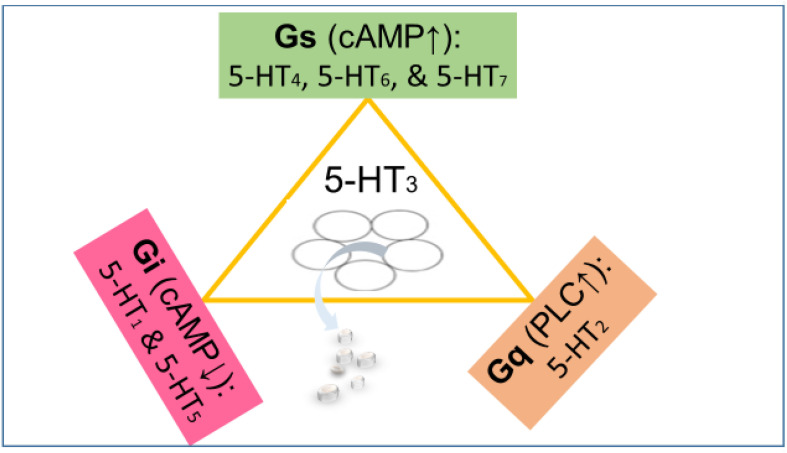
The 5-HT_3_ receptor (center) is an excitatory cation channel (mainly Na^+^ and Ca^2+^), belonging to the cys-loop superfamily of ligand-gated ion channels closely related by homology to the nicotinic acetylcholine receptor. All other serotonin receptors are G-protein-coupled (GPCRs). The 5-HT subtypes 4, 6, and 7 are coupled to stimulatory G proteins (G_s_) responsible for increasing the cyclic AMP concentration. The subtypes 1 and 5 are coupled to inhibitory G proteins (G_i_) responsible for lowering the cyclic AMP concentration. The 5-HT_2_ receptors couple to G_q_ proteins activating phospholipase C (PLC) and ultimately increasing Ca^2+^ concentration (Kaumann and Levy, 2006). The effect of the activation of various serotonin receptors depends not only on the G protein they are coupled to but also on their localization. For instance, a serotonin receptor coupled to G_s_ localized on a GABAergic neuron will enhance inhibition, while a serotonin receptor coupled to G_i_ localized on a GABAergic neuron will reduce inhibition (inhibition of inhibition).

Intravenous administration of 5-HT lowers the heart rate. Since 5-HT is barely able to cross the blood–brain barrier, this is likely an effect in the periphery. The intra-cerebro-ventricular injection (rat) of 5-HT has minor effects on the mean arterial blood pressure but produces a decrease in heart rate (Sévoz-Couche et al., 2000; Villalon and Centurion, 2007; Davisson et al., 2014). However, 5-HT can also cause generalized sympatho-excitation by stimulation of receptors at the site of sympathetic control (rostral ventrolateral medulla) and of receptor-mediated catecholamine release from adrenomedullary chromaffin cells. Neither systemic application nor intra-cerebro-ventricular injection allows identification of the specific receptor subtype involved (Figure 1).

## Serotonin Receptors and Vagal Output

### 5-HT_1A_ Receptors

5-HT_1A_ receptors (G_i_) inhibit adenyl cyclase as their principal signaling mechanism (Kaumann and Levy, 2006). They are located both pre-synaptically and post-synaptically (Stahl, 2015; Svob Strac et al., 2016). Pre-synaptic 5-HT_1A_ auto-receptors located on cell bodies, when stimulated, lead to inhibition of firing of 5-HT neurons and are key components of a negative feedback loop (inhibitory auto-receptors), while pre-synaptic hetero-receptors located on GABAergic neurons reduce neurotransmitter release. By blocking 5-HT_1A_ auto-receptors at doses that are selective for them over post-synaptic 5-HT_1A_ receptors, it is possible to disinhibit 5-HT release. Stimulation of post-synaptic 5-HT_1A_ receptors on GABAergic neurons leads to hyperpolarization and reduced inhibition.

Adding complexity, the 5-HT_1A_ functions as a **hub receptor** in a number of iso- and hetero-receptor dimerizations (Borroto-Escuela et al., 2017). Receptor–receptor interaction (cross talk inhibition) takes place in the 5-HT iso-receptor complexes described (5-HT_1A_-5-HT_7_ and 5-HT_1A_-5-HT_2A_; Renner et al., 2012; Borroto-Escuela et al., 2016, 2017).

Present knowledge indicates that activation of 5-HT_1A_ hetero-receptors enhances vagal activity by disinhibition of glutamatergic neurons [reduction of (inhibitory) GABA release]. Transgenic mice overexpressing 5-HT_1A_ receptors show prolonged episodes of bradycardia, and 5-HT_1A_ agonists induce bradycardia (Ramage, 1990; Jordan, 2005; Audero et al., 2008; Ramage and Villalon, 2008; Restrepo et al., 2010). 5-HT_1A_ receptor agonists produce miosis in humans (Yu et al., 2004). Measurement of pupil size seems to provide a valuable and sensitive index of 5-HT_1A_ receptor function (Fanciullacci et al., 1995).

5-HT_1A_ gene knockout animals showed increased fear and sympatho-activation under experimental conditions (Klemenhagen et al., 2006).

In conclusion, stimulation of 5-HT_1A_ receptors causes central sympatho-inhibition and an increase in cardiac vagal drive (Ramage, 1990).

### 5-HT_2A_ Receptors

5-HT_2A_ receptors [G_q_; activation of phospholipase C→inositol triphosphate (IP_3_↑) and diacylglycerol (DAG↑)] are expressed widely throughout the CNS and periphery (Hoyer et al., 2002). This is the main excitatory receptor subtype among the metabotropic 5-HT receptors. The receptor was first noted for its importance as a target of serotonergic psychedelic drugs such as LSD; later, it came back to prominence, because it was also found to be mediating, at least partly, the action of many antipsychotic drugs. Age-related reduction in the density of 5-HT_2A_ receptors is correlated with cognitive decline (Hasselbalch et al., 2008), and 5-HT_2A_ receptors are decreased in the prefrontal cortex of patients with Alzheimer’s disease (Lorke et al., 2006). In the periphery, it is highly expressed in platelets, cardiovascular system, fibroblasts, and neurons of the peripheral nervous system. Calcium entry through glutamate responsive NMDA channels subsequent to 5-HT_2A_ receptor activation dramatically affects both pre-synaptic and post-synaptic excitability of neurons in the DVN (Huang and Pickel, 2003; Svob Strac et al., 2016). Jordan assigns 5-HT_2A_ receptors a vagal activator effect (Jordan, 2005). In contrast, others expressed the view that activation of 5-HT_2A_ leads to inhibition of parasympathetic synaptic transmission (Chang et al., 2017).

### 5-HT_2B_ Receptors

5-HT_2B_ receptors (G_q_) are located both centrally and in the periphery. Agonists have been associated with endocardial fibrous tissue proliferation and (nor-fenfluramine) valvulopathy (Zanettini et al., 2007; Andersohn and Garbe, 2009); antagonists lack (up to now) a clear therapeutic application. While Jordan (2005) ascribes 5-HT_2B_ receptors a role in vagal activity, this does not appear to be prominent. Nevertheless, in NTS neurons receiving vagal afferent inputs, using ligands selective for the different 5-HT_2_ receptor subtypes, it was observed that activation of 5-HT_2A_ and 5-HT_2B_ receptors had predominantly excitatory effects while activation of 5-HT_2C_ receptors predominantly reduced neuronal firing.

### 5-HT_2C_ Receptors

5-HT_2C_ receptors (G_q_) are structurally similar to 5-HT_2A_ receptors, and the two coexist in many brain regions and on the same neurons. Functionally, 5-HT_2A_ and 5-HT_2C_ are mostly antagonists. They play opposing facilitative and inhibitory roles. 5-HT_2C_ activation inhibits neurotransmitter (dopamine) release. Feeding, social interaction, sexual activity, and drugs (caffeine, nicotine, amphetamine, morphine, cocaine) all induce dopamine release, which is subject to inhibition by 5-HT_2C_. 5-HT_2C_ activation inhibits NTS neurons (vagal activity; Sévoz-Couche et al., 2000; Jordan, 2005).

### 5-HT_3_ Receptors

5-HT_3_ receptors are the only ionotropic serotonin receptors. 5-HT_3_ receptors are located (mainly) on sensory vagal nerve endings and play a vital role for vagal afferent input from the gastrointestinal tract, lungs, and heart. The central terminals of vagal afferents exhibit 5-HT_3_ receptors that function to increase glutamatergic synaptic transmission to second-order neurons of the NTS within the brainstem (Browning, 2015). Experimental compounds with 5-HT_3_ blocking properties increase the heart rate by decreasing vagal afferent input and efferent output; this is compatible with data showing that 5-HT_3_ receptors excite vagal afferent neurons by a glutamate-dependent mechanism (Jordan, 2005; Ramage and Villalon, 2008). Blockade of these receptors by 5-HT_3_ antagonists (setrons) is used clinically for control of emesis (Svob Strac et al., 2016).

### 5-HT_4_ Receptors

5-HT_4_ receptors (G_s_; couple positively to adenylyl cyclase) control acetylcholine release; 5-HT_4_ antagonists have been proposed to treat an overactive bladder (Brudeli et al., 2013) while agonists (-pride) are gastro-kinetic agents. For a number of years (until its removal from the market due to concerns related to QT prolongation), cisapride was (more or less) successfully used to treat hiccups as part of a combination therapy with baclofen or gabapentin and omeprazole (Petroianu et al., 1997; 1998; 2000; 2004).

### 5-HT_5_ Receptors

5-HT_5_ receptors (G_i_ protein coupled) are virtually unexplored due to lack of selective ligands (Pithadia and Jain, 2009).

### 5-HT_6_ Receptors

5-HT_6_ receptors are G_s_ protein coupled and mediate excitatory neurotransmission. 5-HT_6_ receptors are expressed almost exclusively in the brain. Despite the 5-HT_6_ receptor having a functionally excitatory action (G_s_), it is largely co-localized with GABAergic neurons and produces an overall inhibition of brain activity (Yun and Rhim, 2011).

More recently, it was recognized that 5-HT_6_ receptors modulate primarily GABA and glutamate levels, modulating the secondary release of other neurotransmitters (Khoury et al., 2018). Most interestingly, it was recently demonstrated that 5-HT_6_ receptor antagonism reduces defecation in rats (Hagsäter et al., 2019). This finding suggests an involvement of this receptor in the control of parasympathetic activity.

### 5-HT_7_ Receptors

5-HT_7_ receptors (G_s_ protein coupled) are expressed both centrally and in the periphery (Svob Strac et al., 2016). Many—if not all—atypical antipsychotic drugs are also antagonists at this receptor. Defining the influence of this receptor on the vagus nerve is difficult, due to both species differences and the lack of selective agonists and antagonists. There is indication that 5-HT_7_ receptor protein is localized on vagal nerve fibers and that 5-HT_2_ and 5-HT_7_ receptors have opposite effects on vagal activity (García-Pedraza et al., 2017). Initial studies indicate that, in rodents, central 5-HT_7_ receptors play a facilitatory role in the reflex activation of vagal outflow to the heart (Kellett et al., 2005) and that blocking either 5-HT_1A_ or 5-HT_7_ receptors attenuates bradycardias (i.e., increases heart rate), indicating that both subtypes have the ability to activate the vagus nerve (Jordan, 2005; Ramage and Villalon, 2008). It was speculated that vagus activation was mediated by 5-HT_7_ receptors located in the NTS (Jordan, 2005; Kellett et al., 2005). In contrast, García-Pedraza et al. (2017) report that 5-HT_7_ receptor activation suppresses the vagally induced bradycardia, suggesting the opposite, i.e., an inhibitory role of 5-HT_7_ receptors upon vagal activity. Moreover, the same group could demonstrate that 5-HT_7_ activation also stimulates the sympathetic outflow (García-Pedraza et al., 2013). Hernández-Abreu et al. (2020) reported that blockade of 5-HT_2_ receptors uncovers 5-HT_7_ receptors’ ability to inhibit the sympathetic drive in pithed rats, involving hyperpolarization due to the opening of ATP-sensitive K^+^ channels.

Complexity is added by 5-HT_1A_-5-HT_7_ co-expression and heterodimer formation. Hetero-dimerization (cross talk inhibition) decreases the ability of the 5-HT_1A_ receptor to induce hyperpolarization (Renner et al., 2012).

### Summary

**FIGURE 2:**
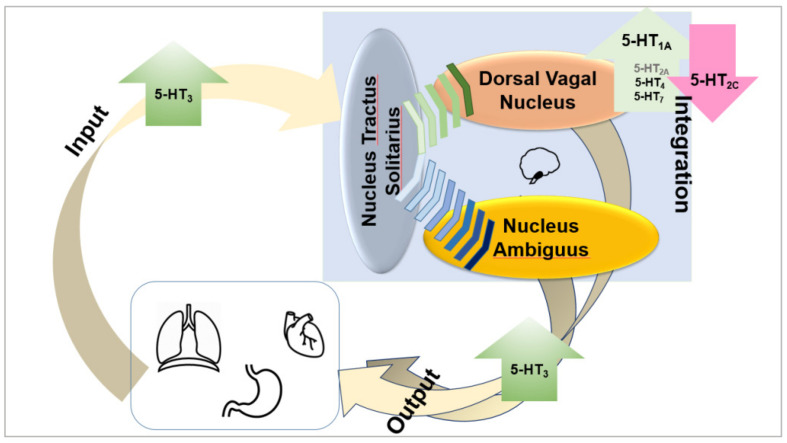
Taken together, it appears that the ability to increase vagus (efferent) output is associated with 5-HT_1A_, 5-HT_3_, and 5-HT_4_ agonists and with 5-HT_2C_ antagonists. The role of 5-HT_7_ and 5-HT_2A_ receptors is not clearly established, but it appears possible that they also enhance vagal output.

Taken together, it appears that the ability to increase vagus (efferent) output is associated with 5-HT_1A_, 5-HT_3_, 5-HT_4_, and possibly 5-HT_7_ agonists and with 5-HT_2__C_ antagonists. The role of 5-HT_2__A_ receptors is not clearly established (Figure 2).

## Purpose of the Work

Vagal mechanisms are operational in the occasional (transient) hiccup and most probably also in chronic singultus. Serotonergic neurotransmission is intricately related to vagal activity. Modulation of serotonergic neurotransmission influences vagal activity, offering possible explanations for the facilitation of singultus (vagal inhibition) by some drugs as well as for the ability of other compounds to suppress hiccups (vagal activation). We will discuss the effects of drugs that either stimulate or block 5-HT receptors upon vagal activity and hiccups. We hope the review will add to the understanding of the phenomenon singultus and possibly trigger a rethinking of the underlying biology of this condition.

### Serotonin 5-HT_1A_ Agonists and Singultus

Activation of 5-HT_1A_ receptors enhances vagal activity; therefore, 5-HT_1A_ agonists could be useful in the control of chronic hiccups.

### Flibanserin

Flibanserin (approved for the treatment of premenopausal women with hypoactive sexual desire disorder) acts as a full agonist of the 5-HT_1A_ receptor and, with lower affinity, as an antagonist of the 5-HT_2A_ receptor (Borsini et al., 2002). To our knowledge, no effect of flibanserin on singultus has been reported.

### Anxiolytic Azapirones

**Tandospirone** was successfully employed for the treatment of intractable hiccups (Takahashi et al., 2004). However, other mechanisms and receptors may also come into play, since a major metabolite of tandospirone, 1-(2-pyrimidinyl)-piperazine (1-PP), is a centrally acting α_2_-adrenergic antagonist (Blier et al., 1991; Onizuka et al., 2002). Central α_2_ antagonism increases 5-HT release and availability (Scheibner et al., 2001). Therefore, the central α_2_-adrenergic antagonist **mirtazapine** (atypical antidepressant), can be considered an indirect 5-HT_1A_ receptor agonist. By additionally antagonizing 5-HT_2_ and 5-HT_3_ receptors (Anttila and Leinonen, 2001), thereby funneling most of its action to the 1A subtype, mirtazapine mainly activates the 5-HT_1A_ receptor; it has also been successfully used for the treatment of intractable singultus (de Boer et al., 1996; Chung et al., 2008).

In contrast, no beneficial effect upon singultus has been reported for **buspirone,** a tandospirone-like drug, which is a weaker partial 5-HT_1A_ agonist that has been available for nearly 30 years and is widely used. It has even been associated—albeit very rarely—with causing hiccups (<1/1000 patients; Silverman et al., 2014; Healthy Place, 2017).

However, these differential effects of tandospirone and buspirone upon singultus may be due to differences in efficacies between these two compounds. Tandospirone and buspirone have similar affinities for the 5-HT_1A_ receptor (≈20–30 nM), but different efficacies. Buspirone has an efficacy (E_max_) of 20–50% at this receptor (as compared with 5-HT), while tandospirone has a higher E_max_ of 80%, closer to that of a full agonist (Yabuuchi et al., 2004; Sumiyoshi et al., 2007).

Typical (haloperidol) and atypical (aripiprazole, olanzapine, risperidone, and brexpiprazole) **antipsychotics** also interact with the 5-HT_1A_ receptor. **Aripiprazole** is a partial 5-HT_1A_ agonist with comparable if not higher affinity (≈1–10 nM) than the spirones (5-HT_1A_ partial agonists), its efficacy being similar to tandospirone (E_max_ = 70%) (Shapiro et al., 2003; Davies et al., 2004).

In contrast, **olanzapine**, **risperidone,** and **haloperidol** have much lower affinities for this receptor (two orders of magnitude) and comparative efficacy (E_max_) in the low negative values (−15, −20, and −10, respectively), indicating antagonist/inverse agonist profiles (Newman-Tancredi et al., 2005).

**Aripiprazole** shows the strongest association of any antipsychotic drugs with hiccup induction. In contrast, olanzapine seems to be a successful antipsychotic agent in the context of singultus suppression (Alderfer and Arciniegas, 2006; Rizzo et al., 2014). There is an abundance of reports on hiccups associated with aripiprazole treatment (Behere et al., 2007; Ginsberg, 2007; Ray et al., 2009; De Filippis et al., 2015; Sakalli Kani et al., 2015; Caloro et al., 2016; Kutuk et al., 2016) and on persistent hiccups associated with switching antipsychotic treatment from risperidone to aripiprazole (Yeh, 2011), from a typical antipsychotic drug of the thioxanthene class to aripiprazole (Duvarci and Yilmaz, 2013), and from olanzapine to aripiprazole (Hori and Nakamura, 2014). In a patient treated with aripiprazole, singultus persisted, despite trials of metoclopramide and chlorpromazine; remission of hiccups occurred after discontinuation of aripiprazole (Silverman et al., 2014).

**Pindolol,** a beta blocker with nanomolar affinity at 5-HT_1A_ receptors (Ki ≈ 10–30 nM) exhibits antagonist properties showing preferential action on somatodendritic 5-HT_1A_ auto-receptors with an efficacy of ≈20% relative to the endogenous agonist. Despite the functional antagonist profile, the drug to our knowledge has not been associated with hiccup induction (Celada et al., 2004).

Aripiprazole, buspirone, pindolol, and tandospirone, having similar effects upon the 5-HT_1A_ receptor, but quite different effects on singultus, do not provide support to the assumption that 5-HT_1A_ receptors play a pivotal role in the pathogenesis of hiccups. Equally unsupportive is the finding that inverse agonists/antagonists such as olanzapine do not seem to induce hiccups and, on the contrary, can be quite useful in treating them.

Takahashi et al. (2004) suggested that 5-HT_1A_ agonists suppress hiccups by inhibiting phrenic nerve activity, while Silverman et al. (2014) proposed that 5-HT_1A_ partial agonists (functional antagonists) promote singultus by enhancing phrenic nerve motor activity.

**Chlorpromazine** (Ki ≈ 840 nM), for all practical purposes, has no effect at the 5-HT_1A_ receptor (Yonemura et al., 1998).

### 5-HT_2A_ Antagonists and Hiccups

Therapeutic efficacy of atypical antipsychotics and their metabolites depends on their high affinity (single digit nanomolar Ki) for and antagonist activity at this receptor subtype. As elaborated above, atypical antipsychotics can either induce or suppress hiccups, due to their affinity to other 5-HT receptor subtypes. With 5-HT_2A_ antagonism being a class-defining property of atypical antipsychotics, the influence of the 5-HT_2A_ receptor upon singultus pathogenesis can therefore not be easily evaluated. For comparison, chlorpromazine has a Ki ≈ 10 nM for this receptor.

### 5-HT_2C_ Agonists and Hiccups

Most typical (including chlorpromazine) and atypical antipsychotics are antagonists or inverse agonists at this receptor. In contrast, the previously mentioned atypical aripiprazole binds with nanomolar affinity at 5-HT_2C_ receptors (Ki ≈ 15–180 nM) and exhibits partial agonist properties with an efficacy Emax ≈ 80% relative to the endogenous agonist. **Lorcaserin**, marketed for weight loss, is the only selective 5-HT_2C_ receptor agonist clinically available (Ki ≈ 15 nM; Emax ≈ 40%). There are no reports regarding lorcaserin and hiccup. **Pimavanserin**, marketed for Parkinson’s psychosis, is a 5-HT_2A_ and 5-HT_2C_ receptor antagonist (Ki ratio ≈ 1: 40; Stahl, 2016b). There are no reports regarding pimavanserin and singultus.

### 5-HT_3_ Antagonists and Hiccups

Antagonists **(-*setrons*)** are potent and highly selective competitive inhibitors with negligible affinity for other receptors. They are rapidly absorbed and penetrate the blood–brain barrier easily. Antiemetic efficacy results from a simultaneous action at peripheral and central 5-HT_3_ receptors.

Blockage of 5-HT_3_ receptors in the periphery reduces the activity of vagal afferents and would thus decrease efferent output; blocking 5-HT_3_ receptors centrally would also reduce efferent output. A single anecdotal mentioning of a negative impact of ***setrons*** on a patient with chronic hiccup has been published in 1992 (Petroianu and Brunnengraber, 1992). There are numerous anecdotal reports in the non-scientific literature claiming that setrons cause hiccups (Wilkes, 2007; Kantrowitz, 2009; MylanPharmaceuticals, 2017; Pharmacorama, 2017). Chlorpromazine has a very low affinity for this receptor (Yonemura et al., 1998). Taken together, these data suggest that 5-HT_3_ antagonists may facilitate singultus.

### 5-HT_7_ Antagonists and Hiccups

Initial studies suggest that activation of 5-HT_7_ receptors increases efferent vagal activity (Jordan, 2005; Kellett et al., 2005); more recent reports, however, indicate the opposite (García-Pedraza et al., 2017). Most antipsychotics (chlorpromazine, clozapine, risperidone, ziprasidone, paliperidone, pimozide, and amisulpride) are antagonists at the 5-HT_7_ receptors.

Nishikawa and his colleagues reported on a patient with intractable hiccups where haloperidol failed to provide relief, while in contrast, **risperidone** completely abolished the singultus shortly after administration (Nishikawa et al., 2015). While both haloperidol and risperidone are antagonists with comparably low nanomolar affinity at D_2_ receptors, only risperidone blocks 5-HT_2C_ receptors and has an affinity, at least one order of magnitude higher (lower Ki) than haloperidol at 5-HT_2A_ and 5-HT_7_ receptors (Roth et al., 1994; Amato et al., 2015). The authors conclude that the ability of risperidone to suppress hiccups versus the failure of haloperidol to do so indicates that the serotonergic system may play a role in the pathophysiology of some hiccup forms (Nishikawa et al., 2015). Notwithstanding such therapeutic successes, there are also case reports of risperidone inducing singultus (Cheng and Tsai, 2015).

**Pimozide** acts as an antagonist at D_2_-like receptors and the 5-HT_7_ receptor; it has the highest affinity of all the typical antipsychotic agents tested for the 5-HT_7_ receptor (Ki < 1 nM; Roth et al., 1994). Pimozide is anecdotally reported to be clinically used to control not only nausea and vomiting but also intractable hiccups (DrugInfoSys, 2016). However, there are also reports on pimozide causing hiccups (Merck, 2017).

**Amisulpride**, a benzamide antagonist of the dopamine D_2_ and D_3_ receptors and an antagonist of the 5-HT_2B_ and 5-HT_7_ receptors has been reported to induce singultus in a schizophrenic patient not sufficiently controlled on paliperidone (Cheng and Tsai, 2015). Paliperidone (9-OH-risperidone) has a receptor profile very similar to risperidone, with the difference of a lower affinity (antagonist) at the 5-HT_1A_ receptor. Compared with amisulpride, the affinity of the two for 5-HT_7_ is very similar (≈10 nM). Amisulpride has, however, no effect on other 5-HT receptors except 5-HT_2B_ (15 nM; Abbas et al., 2009).

Aripiprazole, while commonly described as a partial agonist at 5-HT_7_, has a low intrinsic activity (Emax ≈ 2%), and hence is a functional antagonist of this receptor.

In summary, the observed pharmacological effects of medications upon hiccup can be attributed to a variety of receptors, including the 5-HT_7_ type. As long as there are no selective ligands of the 5-HT_7_ receptor that are clinically available, it is difficult to draw conclusions about the role of this receptor upon hiccup. Available data, however, suggest an influence of 5-HT_7_ receptors upon singultus pathogenesis and therapy.

## Conclusion and Outlook

The overlap between maneuvers used to terminate paroxysmal supraventricular tachycardia, a not uncommon cardiac arrhythmia, and those employed to terminate bouts (paroxysms) of hiccups is striking. It suggests that activation of efferent vagal fibers can be therapeutic in both instances. While coincidence is obviously not proof of causality, it warrants nevertheless further investigations.

Taken together, it appears that the ability to increase vagus (efferent) output is associated with 5-HT_1A_, 5-HT_3_, and 5-HT_4_ agonists and with 5-HT_2C_ antagonists. The role of 5-HT_7_ receptors is not clearly established, but it appears possible that they also enhance vagal output.

Chlorpromazine does not display the ideal required profile for a vagus activator (Table 1).

**TABLE 1:** Chlorpromazine, while without effect at 5-HT_1A_, 5-HT_3_, and 5-HT_4_ receptors, is a 5-HT_2A_, 5-HT_2C_, 5-HT_6_, and 5-HT_7_ antagonist and thus theoretically—at least from a serotonergic perspective—does not display the ideal required profile for a vagus activator. Affinities for the dopaminergic receptors are also provided.

	5-HT (K*i* nM); antagonist						
	1A	2A	2C	3	4	6	7
Chlorpromazine	800+	10–15	15–25	600+	Not available	5	20

	**Dopamine (K*i* nM); antagonist**						
	**1**	**2**	**3**	**4**	**5**		

Chlorpromazine	6–96	2–12	2–10	8–56	Not available		

*(Kuoppamäki et al., 1995; Yonemura et al., 1998; Glennon, 2003; Kusumi et al., 2015).*

A review of the various drug actions does not warrant a definitive conclusion at this time. While painfully aware of the limitations of comparing receptor affinities/intrinsic activity values—even more so when obtained from different sources using different methodologies—and inferring biological effects based on such data, it is still the only practical option available (de Bartolomeis et al., 2015; Das et al., 2016; Stahl, 2016a). We nevertheless hope our work might add to the understanding of the phenomenon singultus and possibly trigger a rethinking of the underlying biology of this condition.

## Author Contributions

GP and DL drafted the manuscript and both approved the final version.

## Conflict of Interest

The authors declare that the research was conducted in the absence of any commercial or financial relationships that could be construed as a potential conflict of interest.
